# IPLG: Intent-Propagated Lane Graph for Multimodal Trajectory Prediction at Urban Intersections

**DOI:** 10.3390/s26144527

**Published:** 2026-07-16

**Authors:** Yibo Xu, Yajie Zou, Yichuan Peng, Amin Moeinaddini, Yubin Chen

**Affiliations:** 1 Key Laboratory of Road and Traffic Engineering of Ministry of Education, Tongji University, Shanghai 201804, China; 2310839@tongji.edu.cn (Y.X.);; 2 Department of Civil and Environmental Engineering, Amirkabir University of Technology, Tehran 1591634311, Iran; 3 College of Transportation and Navigation, Quanzhou Normal University, Quanzhou 362000, China

**Keywords:** trajectory prediction, urban intersection, lane graph, multimodal prediction, intent propagation, topology-constrained decoding, goal diversity

## Abstract

Vehicle trajectory prediction at urban intersections is challenging due to strong maneuver ambiguity, dense agent interactions, and complex lane branching structures. Existing methods frequently suffer from mode collapse or fail to provide sufficient coverage across all competing exit branches, limiting their reliability in safety-critical scenarios. This paper proposes IPLG, an Intent-Propagated Lane Graph framework for multimodal vehicle trajectory prediction at UAV-observed urban intersections. The proposed framework addresses two core challenges: the semantic ambiguity of lane nodes at divergence points under competing agent intentions, and the tendency of standard decoders to generate trajectories that deviate from valid lane regions. To this end, IPLG introduces a closed-loop interaction module that propagates agent intent cues into the lane graph and along its topology, a topology-aware diversified goal selection strategy that suppresses goal clustering across competing exit branches, and a residual decoder that enforces local lane consistency at each prediction step. Experiments on the XJROAD dataset show that IPLG achieves consistent performance across multiple evaluation metrics, with noticeable improvements in Miss Rate and Off-road Rate. Additional validation on the public nuPlan dataset demonstrates that the proposed model can generate plausible trajectories across different intersection geometries, further supporting its generalizability across diverse road structures and driving scenarios.

## 1. Introduction

Accurate trajectory prediction of surrounding vehicles is essential for autonomous driving, especially in urban intersections where traffic behavior is highly interactive, topologically constrained, and inherently multimodal—that is, a single agent may follow any of several plausible future trajectories depending on its intent [1]. In these scenarios, a target vehicle may plausibly go straight, turn left, turn right, or yield under similar short-term observations. Such uncertainty is intensified by dense interactions among neighboring agents and by the complex branching structure of lane topology [2]. Therefore, trajectory prediction at intersections remains a challenging task for downstream planning and decision-making modules [3].

Recent trajectory prediction research has increasingly focused on richer scene representations and more effective interaction modeling, reflecting a broader shift from history-based prediction toward map-aware, graph-based, Transformer-based [4,5,6], and generative frameworks [7]. Early methods mainly relied on historical agent states and neglected map context [1]. Later approaches incorporated high-definition maps through rasterized bird’s-eye-view representations [8] or vectorized lane-based encodings [9]. Compared with rasterized representations, lane-graph-based methods [10] provide a more compact and topology-aware description of the road environment, and are better suited to preserving directional semantics and lane connectivity. However, most existing methods treat the lane graph as a static context source: lane features are encoded once from map geometry and then aggregated into agent representations, while agent motion cues are not fed back to update lane-node semantics [11]. This one-directional flow is particularly problematic at intersection divergence points. The semantic role of a lane node—whether it leads to a left turn, a right turn, or a straight-through movement—cannot be determined from geometry alone, but depends on the intentions of nearby agents [12]. Without agent-to-lane feedback, divergence-point nodes remain geometrically encoded but intention-agnostic, limiting the model’s ability to distinguish among competing exit branches during goal inference.

**Figure 1:**
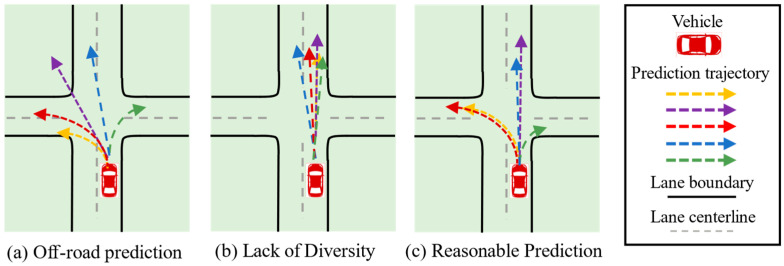
Representative multimodal maneuver scenarios at urban intersections.

A second challenge concerns multimodal goal prediction and diversity. Although goal-guided multimodal prediction has shown clear advantages over simple regression-head or latent-sampling approaches [13], goal clustering and mode collapse still frequently occur in high-uncertainty scenarios [14]. High-probability goals tend to concentrate on dominant branches, weakening the coverage of competing exit intentions [15]. These issues are especially severe at urban intersections, where multiple feasible exit branches coexist within a compact spatial range and competing maneuver intentions are difficult to distinguish from short-term observations alone. Figure 1 illustrates several representative multimodal maneuver scenarios at urban intersections, highlighting the strong branch ambiguity and diversity requirements faced by prediction models.

To address these challenges, this paper proposes an Intent-Propagated Lane Graph framework with Topology-Constrained Residual Decoding for multimodal vehicle trajectory prediction at urban intersections. The framework contains three main components. First, an Intent-Propagated Interaction Flow (IPIF) models the closed-loop dependency between agents and the lane graph through four sequential stages: N2A aggregates road-context information from lane nodes into agent representations; A2A captures social dynamics among neighboring agents; A2N injects agent kinematic cues back into lane nodes, resolving the semantic ambiguity of divergence-point nodes whose topological role depends on surrounding agent intent; and N2N propagates the resulting intent-aware features along lane topology, enabling downstream branch nodes to encode dynamic intention context before goal selection. Second, a topology-aware goal selection strategy operates on the topology-propagated intent-aware lane features to select a diverse and feasible set of K goals while reducing goal clustering. Third, a Topology-Constrained Residual Decoder (TCRD) generates goal-conditioned trajectories through an initial Gated Recurrent Unit (GRU) pass followed by a residual refinement step using nearby intent-aware lane-node context, improving lane alignment and reducing off-road violations.

The main contributions of this study are summarized as follows:

(1) We propose IPLG, an intent-propagated lane-graph framework with topology-constrained residual decoding for multimodal trajectory prediction at urban intersections, jointly addressing goal diversity and trajectory topological consistency in complex branching scenarios.
(2) We introduce IPIF, a four-stage interaction module that propagates agent intent cues from agents to lane nodes and further along lane topology through N2N reasoning: N2A aggregates road context into agent representations; A2A captures social dynamics among agents; A2N injects agent kinematic cues back into lane nodes, resolving semantic ambiguity at divergence points; and N2N propagates the resulting intent-aware features along lane topology, enabling downstream branch nodes to encode dynamic intention context before goal selection.
(3) We propose a topology-aware diversified goal selection strategy that combines entropy regularization with combinatorial optimization, enabling selected goals to cover competing exit branches while suppressing goal clustering, without requiring manual anchor design.
(4) We propose TCRD, which refines initial goal-conditioned GRU predictions using local intent-aware lane-node context at each prediction step, improving lane alignment and reducing off-road violations without constraining trajectory diversity.

The rest of this paper is organized as follows. Section 2 reviews related work on scene representation, interaction modeling, and multimodal trajectory prediction. Section 3 introduces the proposed framework. Section 4 presents the experimental settings and results. Section 5 presents the conclusion.

## 2. Related Work

### 2.1. Scene Representation with Lane Graphs

Accurate trajectory prediction depends heavily on an effective representation of both dynamic agents and structured road environments. Early prediction methods mainly rely on the historical states of agents while neglecting map context [1]. More recent studies incorporate high-definition (HD) maps through rasterized bird’s-eye-view representations processed by convolutional neural networks (CNNs) [8], but raster-based methods suffer from information loss during rendering and are less effective in capturing fine-grained lane topology and directional semantics [16].

To better preserve road geometry and connectivity, vectorized representations have become increasingly popular. VectorNet [9] encodes both road structures and agent trajectories as compact polyline sets and processes them through a hierarchical graph neural network. Building on this idea, lane-graph-based methods such as LaneGCN [10] model lane centerlines as directed graphs, where nodes correspond to short centerline segments and edges describe successor, predecessor, and proximal relationships. LaneGCN further introduces multi-scale graph convolutions with skip connections to aggregate topology over varying spatial ranges, providing a compact, topology-aware description of traffic scenes that helps ensure predicted trajectories remain aligned with valid lane structures.

Recent work has further emphasized the role of lane segments as intention-related semantic units rather than purely geometric carriers. A distributed lane-centric representation [17] uses individual lane segments as the basic units of scene understanding, highlighting the importance of structured lane-level semantics in long-horizon prediction. Our work also adopts a lane-graph representation, but differs from existing methods in that lane nodes are enriched with dynamic agent intent cues rather than encoded purely from static map geometry, enabling more discriminative representations at intersection divergence points.

### 2.2. Interaction Modeling for Multimodal Trajectory Prediction

Another key challenge lies in modeling interactions among traffic participants and between agents and the surrounding scene. Early studies rely on social pooling mechanisms [18] or handcrafted interaction functions, which are limited in preserving fine-grained spatial relationships. Attention-based methods [19] later became the dominant paradigm, allowing models to adaptively aggregate relevant information from neighboring agents and map elements. Related attention mechanisms have also been used to model social interactions in crowd scenarios [20], while recent studies have examined the interpretability of attention-based multimodal prediction [21]. More recent studies have applied Transformer architectures [22] and efficient sequence modeling methods [23] to capture spatiotemporal dependencies across agent–agent and agent–scene dimensions.

With the increasing use of HD maps, several studies have explored interaction modeling on graph-structured scenes. PGP [24] aggregates scene context through lane-graph traversals conditioned on a learned behavioral policy. Chen et al. [25] introduce an agent-node attention mechanism with three interaction stages, namely N2A, A2A, and A2N, and show that feeding agent information back into lane nodes improves intention inference and trajectory generation. A historical prediction attention network [26] hierarchically models agent–scene and agent–agent dependencies across consecutive prediction frames, further highlighting the importance of multi-step hierarchical reasoning. Intersection-specific methods [27] further highlight the distinct challenges of signal-controlled urban scenarios, where compact branch geometry and signal timing impose additional constraints on trajectory modeling.

Despite these advances, interaction modeling at urban intersections remains challenging. Compared with ordinary road segments, intersections contain denser conflict relationships, stronger topological branching, and more uncertain maneuver choices. The proposed framework addresses these challenges through an IPIF that models agent–lane and lane–lane dependencies in a unified closed-loop manner. An enriched 8-dimensional kinematic state vector including lateral offset and acceleration enables more discriminative intent cues to be injected into lane nodes at branching points through A2N. The subsequent N2N stage further propagates these intent semantics along lane topology, allowing downstream branch nodes to inherit intention context from upstream divergence points—a capability not present in prior agent–lane interaction methods.

### 2.3. Goal-Guided Multimodal Prediction and Diversity Enhancement

Multimodal prediction aims to generate multiple plausible futures rather than a single deterministic trajectory. Early multimodal models relied on multiple regression heads [8] or latent-variable sampling [13], which frequently suffer from mode collapse—generating only a few dominant modes or producing trajectories that violate road constraints [14]. To address this problem, goal-guided prediction has emerged as an effective paradigm. TNT [28] samples goal points along lane centerlines and generates trajectories conditioned on each goal. DenseTNT [29] extends this idea with dense end-to-end goal prediction and offline optimization for goal set selection. These methods significantly reduce the solution space and improve output diversity, but their performance depends heavily on the quality and spatial distribution of the selected goals.

Existing goal-guided methods differ in how they define and select goals. Anchor-based approaches [30] pre-define candidate endpoints along lane centerlines, but manually designed anchors generalize poorly across intersection topologies [14]. Learning-based methods can estimate goal probabilities but tend to concentrate predictions in high-probability regions, leading to densely clustered goals that fail to cover rare maneuvers. Chen et al. [25] address this issue by combining learning-based node probability estimation with maximum-entropy regularization and genetic algorithm optimization, and show that entropy-regularized goal selection can substantially improve trajectory diversity. Recent graph-based goal-conditioned methods [31] further demonstrate the effectiveness of combining lane graph topology with cross-context attention for diverse multimodal prediction. In parallel, plan-conditioned methods have explored the use of grid-based plans to guide trajectory forecasting in uncertain environments [32].

The proposed framework addresses mode collapse at urban intersections through a topology-aware diversified goal selection module that operates on topology-propagated intent-aware lane features produced by IPIF, enabling the selected goals to cover competing exit branches more effectively than methods based on static lane encodings [10,24]. Unlike prior goal-guided methods that treat encoding and goal selection as independent stages [28,29], the proposed framework tightly couples the IPIF encoder with the goal selection module, providing the goal predictor with richer contextual input that reflects both agent interaction dynamics and lane topology. Related intention-aware intersection methods have also explored trajectory planning or motion prediction from different perspectives. Chen [33] focus on sampling-based trajectory planning with dual-layer probabilistic intention prediction to generate executable trajectories in complex intersections, while Zhang [34] predict intersection vehicle motion by combining turning intention and prior trajectory patterns. Compared with these intention-aware planning or prior-trajectory-based prediction methods, IPLG focuses on multimodal trajectory prediction by using lane-node topology to represent competing intersection branches and by dynamically updating lane-node semantics with surrounding-agent information.

## 3. Proposed Methodology

**Figure 2:**
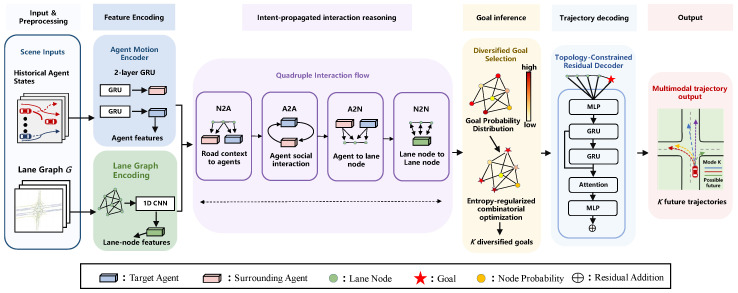
Overall architecture of the proposed framework.

Figure 2 illustrates the overall architecture of the proposed framework. The pipeline consists of four main stages: (1) feature encoding, (2) intent-propagated interaction reasoning, (3) goal inference, (4) topology-constrained trajectory decoding. Historical agent states and lane-graph information are first encoded into compact agent and lane-node features. The IPIF then updates these features sequentially through N2A, A2A, A2N, and N2N interactions. The resulting topology-propagated intent-aware lane features are used for goal probability estimation and diversified goal set optimization. Finally, the TCRD generates K plausible future trajectories.

### 3.1. Problem Formulation

We first formulate the multimodal vehicle trajectory prediction problem. Let A={1,2,…,N} denote the set of N traffic agents observed in a scene. For each agent i its historical motion over $T_{obs}$ time steps is represented as

(1) $$S^{i}=\left[s_{-T_{\mathrm{obs}}+1}^{i},s_{-T_{\mathrm{obs}}+2}^{i},\ldots ,s_{0}^{i}\right],$$

where each state vector is an 8-dimensional kinematic descriptor

(2) $$s_{t}^{i}=\left[x_{t}^{i},y_{t}^{i},v_{t}^{i},a_{t,\tan}^{i},a_{t,\mathrm{lat}}^{i},\omega_{t}^{i},d_{t}^{i},U^{i}\right]$$

Here, $\left(x_{t}^{i},y_{t}^{i}\right)$ denotes the coordinates, $v_{t}^{i}$ the speed, $a_{t,\tan}^{i}$ and $a_{t,\mathrm{lat}}^{i}$ the tangential and lateral accelerations, $\omega_{t}^{i}$ the heading rate, $d_{t}^{i}$ the lateral offset relative to the nearest lane centerline, and $U^{i}$ a categorical agent-type indicator. All agent states are transformed into a target-vehicle-centric coordinate frame, where the target vehicle’s position and heading at t=0 serve as the origin and reference direction.

The road environment is represented as a directed lane graph G=(V,E), where V is the set of lane nodes and E is the set of directed edges. Each node $v_{n}\in V$ corresponds to a short lane centerline segment described by a sequence of *P* geometry poses

(3) $$A_{n}=\left[A_{1}^{n},A_{2}^{n},\ldots ,A_{P}^{n}\right],A_{p}^{n}=(x_{p}^{n},y_{p}^{n},\theta_{p}^{n}),$$

where $\left(x_{p}^{n},y_{p}^{n}\right)$ is the spatial coordinate and $\theta_{p}^{n}$ is the local heading of the *p*-th pose in node $v_{n}$. The edge set E contains three relation types: successor edges $E_{suc}$ encoding longitudinal forward connectivity, predecessor edges $E_{pre}$ encoding backward connectivity, and proximal edges $E_{prox}$ encoding adjacency between neighboring lanes. At intersection divergence areas, a single node may have multiple successor edges, directly encoding the branching topology characteristic of urban intersections.

Given the historical observations ${\left\{S^{i}\right\}}_{i\in A}$ and the lane graph G, the objective of IPLG is to generate *K* plausible future trajectories for the target vehicle over a prediction horizon of $T_{\mathrm{pred}}$ time steps

(4) $$\hat{T}=\left\{{\hat{\tau }}_{1:T_{\mathrm{pred}}}^{\;k}|k=1,2,\ldots ,K\right\},$$

where each trajectory ${\hat{\tau }}_{1:T_{\mathrm{pred}}}^{\;k}=\left[{\hat{\tau }}_{1}^{\;k},{\hat{\tau }}_{2}^{\;k},\ldots ,{\hat{\tau }}_{T_{\mathrm{pred}}}^{\;k}\right]$ is a sequence of predicted BEV positions, with superscript k indexing the predicted mode and subscript t denoting the predicted position of the target vehicle at time step *t*. The generated trajectories are expected to satisfy two conditions simultaneously: (1) prediction accuracy, meaning at least one predicted trajectory should closely match the ground truth; (2) topological consistency, meaning all predicted trajectories should remain compatible with the reachable structure of the lane graph G.

### 3.2. Feature Encoding

The feature encoding stage extracts compact representations from both dynamic agent histories and static lane geometry, providing the input foundation for subsequent interaction reasoning.

#### 3.2.1. Dynamic Agent Encoding

For each agent *i*, the historical state sequence $S^{i}$ is encoded using a two-layer Gated Recurrent Unit (GRU) [35]. Since observations closer to the prediction start time carry stronger predictive signal, the GRU’s gating mechanism naturally emphasizes recent kinematic changes—such as deceleration before a turn or lateral drift toward a lane boundary—making it well suited for capturing short-term intent cues. The encoded agent feature is

(5) $$h^{i}=\mathrm{GRU}(S^{i})\in {\mathbb{R}}^{d_{h}},$$

where $h^{i}$ compresses the full temporal dynamics of agent i’s motion history. The target vehicle embedding is denoted $h_{\mathrm{ego}}$, and the surrounding agent embeddings are collectively denoted $\left\{h_{\mathrm{surr}}^{i}\right\}$.

#### 3.2.2. Lane Node Encoding

Unlike agent trajectories, the poses within a lane node represent local geometric structure rather than temporal evolution, so all poses carry equal importance. Applying a recurrent encoder here risks selectively forgetting certain poses. Instead, we employ a 1D Convolutional Neural Network (1D-CNN) [36] to extract local lane geometry features from each node $v_{n}$

(6) $$a_{\mathrm{node}}^{n}=\mathrm{Pool}(\sigma (\mathrm{Conv}1D(A_{n};\Psi ))),$$

where Ψ denotes learnable convolutional kernels, σ(⋅) is the ReLU activation, and Pool(⋅) is adaptive max-pooling that aggregates local convolutional responses into a fixed-dimensional descriptor. The resulting $a_{\mathrm{node}}^{n}$ captures local lane geometry such as curvature and heading variation.

Each lane node is encoded by the 1D-CNN to preserve local geometric features such as curvature and heading variation. The resulting $a_{\mathrm{node}}^{n}$ serves as the initial node feature for the subsequent IPIF stages. Topological propagation is deferred to the N2N stage within IPIF, after agent kinematic cues have been injected into lane nodes through A2N, ensuring that topology propagation reflects dynamic interaction context rather than static geometric structure alone.

### 3.3. Intent-Propagated Interaction Flow and Goal Inference

**Figure 3:**
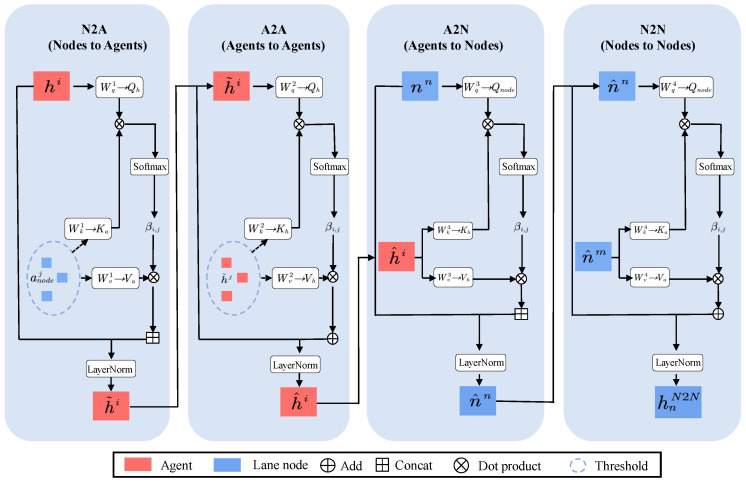
Architecture of the Intent-Propagated Interaction Flow.

This section presents the core interaction reasoning module of the proposed framework. The objective is to transform static lane-node features into topology-propagated, intent-aware representations through a four-stage closed-loop interaction process. As illustrated in Figure 3, the Intent-Propagated Interaction Flow (IPIF) consists of four sequential stages: N2A, A2A, A2N, and N2N. N2A and A2A establish agent-level road-context awareness and social dynamics; A2N feeds agent intent cues back into lane nodes; and N2N propagates the resulting intent-aware features along lane topology, enriching downstream branch nodes before goal inference.

The four interaction stages are not independent attention blocks but form an ordered information-dependency process. Their functional relationships can be summarized as

(7) $$\begin{array}{c} {\tilde{h}}^{i}=N2A\left(h^{i},{\left\{a_{\mathrm{node}}^{n}\right\}}_{n\in N_{l}^{i}}\right),\\ {\hat{h}}^{i}=A2A\left({\tilde{h}}^{i},{\left\{{\tilde{h}}^{j}\right\}}_{j\in N_{a}^{i}}\right),\\ {\hat{n}}^{n}=A2N\left(n^{n},\left\{{\hat{h}}^{i}|n\in N_{l}^{i}\right\}\right),\\ h_{n}^{N2N}=N2N\left({\hat{n}}^{n},{\left\{{\hat{n}}^{m}\right\}}_{m\in N_{r}(n)}\right). \end{array}$$

This ordering reflects a directed dependency among the four representations. N2A first conditions each agent feature on its local lane geometry, ensuring that the subsequent A2A interaction is interpreted under the corresponding road constraints. A2A then incorporates social responses from surrounding agents, producing interaction-aware motion features. A2N feeds these socially refined motion features back into nearby lane nodes, transforming their static geometric representations into agent-conditioned intention representations. Finally, N2N propagates the updated intention information along the directed lane topology. If N2N were applied before A2N, it would propagate only static geometric information and would not convey agent-conditioned branch preferences.

#### 3.3.1. Nodes-to-Agents (N2A)

In the first stage, each agent aggregates road-context information from nearby lane nodes via cross-attention. The agent feature acts as the query, while surrounding lane-node features serve as keys and values. Let $N_{l}^{i}$ denote the set of lane nodes within a predefined distance threshold from agent i. For agent i, the attention weight $\beta_{i,j}$ assigned to lane node $j\in N_{l}^{i}$

(8) $$\beta_{i,j}=\mathrm{soft}\max\left(q_{h}^{i}\cdot k_{a}^{j}\right),$$

where $q_{h}^{i}$ is the query projection of agent feature $h^{i}$, and $k_{a}^{j}$ is the key projection of lane-node feature $a_{\mathrm{node}}^{j}$. The updated agent feature is obtained by concatenating the original encoding with the weighted sum of lane-node values

(9) $${\tilde{h}}^{i}=\mathrm{Concat}\left(h^{i},\sum_{j\in N_{l}^{i}}\beta_{i,j}v_{a}^{j}\right),$$

where $v_{a}^{j}$ is the value projection of $a_{\mathrm{node}}^{j}$. This stage allows each agent representation to incorporate local road-context information and lane-topology constraints, providing the foundation for social interaction modeling in the subsequent A2A stage.

#### 3.3.2. Agents-to-Agents (A2A)

The second stage models social dynamics among road users. Each agent attends to the updated representations of its neighboring agents ${\left\{{\tilde{h}}^{j}\right\}}_{j\in N_{a}^{i}}$, capturing collective behaviors such as car-following, yielding, and gap-acceptance

(10) $${\hat{h}}^{i}=\mathrm{LayerNorm}\left({\tilde{h}}^{i}+\mathrm{Attention}\left({\tilde{h}}^{i},{\left\{{\tilde{h}}^{j}\right\}}_{j\in N_{a}^{i}}\right)\right).$$

The resulting ${\hat{h}}^{i}$ captures both road-context awareness and social dynamics, and serves as the source of intent cues for the A2N stage.

#### 3.3.3. Agents-to-Nodes (A2N)

The third stage closes the agent-to-lane feedback loop by propagating updated agent-motion information back into lane nodes. Vehicle states are mapped onto lane-node semantics in the latent feature space rather than being directly assigned to individual nodes. As defined in Section 3.1, the historical state sequence $S^{i}$ is first encoded by the GRU into the agent feature $h^{i}$. After incorporating local lane context through N2A and interactions with surrounding agents through A2A, the updated feature ${\hat{h}}^{i}$ contains historical motion, road-context, and social-interaction information.

During A2N, $n^{n}=a_{\mathrm{node}}^{n}$ denotes the initial geometric representation of lane node $v_{n}$. The lane-node feature is projected into the query $Q_{\mathrm{node}}$, while the A2A-updated features ${\hat{h}}^{i}$ of nearby agents are projected into $K_{\mathrm{agent}}$ and $V_{\mathrm{agent}}$. The lane-node representation is updated as

(11) $${\hat{n}}^{n}=\mathrm{LayerNorm}\left(n^{n}+\mathrm{Attention}\left(Q_{\mathrm{node}},K_{\mathrm{agent}},V_{\mathrm{agent}}\right)\right),$$

the attention weights determine the contribution of each nearby agent to lane node $v_{n}$. Therefore, $n^{n}$ describes the static lane geometry, whereas ${\hat{n}}^{n}$ additionally encodes the motion and intention context induced by nearby agents. Since $a_{t,\mathrm{lat}}^{i}$, $\omega_{t}^{i}$, and $d_{t}^{i}$ are included in $S^{i}$, different turning or lane-changing tendencies produce different semantic updates for the same geometric lane node.

#### 3.3.4. Nodes-to-Nodes (N2N)

The N2N stage propagates the intent-aware lane-node features ${\hat{n}}^{n}$ produced by A2N along the lane graph topology. For each lane node n, N2N aggregates information from its topological neighbors across three relation types—successor ($E_{\mathrm{suc}}$), predecessor ($E_{\mathrm{pre}}$), and proximal ($E_{\mathrm{prox}}$)—using relation-specific attention weights

(12) $$\beta_{n,m}^{r}=\mathrm{softmax}\left(q_{n}^{n}\cdot k_{n}^{m}\right),$$

where $q_{n}^{n}$ is the query projection of node n, $k_{n}^{m}$ and $v_{n}^{m}$ are the key and value projections of neighboring node *m* under relation $r\in \{E_{\mathrm{suc}},E_{\mathrm{pre}},E_{\mathrm{prox}}\}$, and $N_{r}(n)$ denotes the set of neighbors of node *n* under relation *r*. The updated node feature is

(13) $$h_{n}^{N2N}=\mathrm{LayerNorm}\left({\hat{n}}^{n}+\sum_{r}\sum_{m\in N_{r}(n)}\beta_{n,m}^{r}\cdot v_{n}^{m}\right),$$

N2N propagates dynamically updated intent features that have already been enriched by agent interaction through A2N. As a result, $h_{n}^{N2N}$ encodes lane geometry, topological connectivity, and the evolving intention context induced by neighboring agents. This allows downstream branch nodes—which may not have nearby agents of their own—to inherit intent semantics from upstream divergence-point nodes, improving goal probability estimation across the full lane graph.

To further formalize the intersection-branching semantics, consider a divergence node $v_{n}$ with multiple successor nodes $m\in N_{E_{\mathrm{suc}}}(n)$. Let c denote the traffic-flow context encoded in the A2N-updated lane-node features. Here, c is not an additional manually assigned intention label, but represents the motion and interaction information already contained in ${\hat{n}}^{n}$. For the successor relation $r=E_{\mathrm{suc}}$, the context-conditioned attention weight is written as

(14) $$\beta_{n,m}^{E_{\mathrm{suc}}}\left(c\right)=\frac{\exp\left(q_{n}^{n}(c)\cdot k_{n}^{m}(c)\right)}{\sum_{j\in N_{E_{\mathrm{suc}}}(n)}\exp\left(q_{n}^{n}(c)\cdot k_{n}^{j}(c)\right)},\;\;m\in N_{E_{\mathrm{suc}}}\left(n\right).,$$

The successor set $N_{E_{\mathrm{suc}}}(n)$ is determined by the static lane topology and remains unchanged across different traffic-flow contexts. However, $q_{n}^{n}(c)$ and $k_{n}^{m}(c)$ are derived from agent-conditioned lane-node features and therefore vary with c. Consequently, the same divergence node can assign different semantic importance to its successor branches under different motion intentions. For example, turning-related motion cues increase the attention weight of the corresponding turning branch, whereas straight-going cues increase the weight of the downstream straight branch. These context-dependent branch features subsequently affect the node goal probabilities $\theta_{n}$. Thus, the geometric identity and connectivity of a lane node remain fixed, while its semantic role is dynamically conditioned on the inferred traffic-flow intention.

Although LaneGCN [10] also combines actor-to-lane interaction with lane-graph convolution, its lane propagation primarily serves general actor–map feature fusion. In contrast, the proposed N2N is positioned after the complete N2A–A2A–A2N sequence and operates on agent-conditioned lane-node features. It propagates these intention-related features from locally observed divergence nodes toward downstream branches before goal selection. Therefore, the contribution of N2N lies in its causal position and its task-specific role in connecting agent interaction, branch-level goal inference, and trajectory decoding, rather than in graph propagation alone.

#### 3.3.5. Goal Probability Estimation

Building on the intent-aware lane features, a Multi-Layer Perceptron (MLP)-based scorer estimates the probability of each lane node being the target vehicle’s intended goal. For each candidate node *n*, the fused feature is constructed by concatenating the target vehicle’s updated embedding ${\hat{h}}_{\mathrm{ego}}$ with the node’s intent-aware feature $h_{n}^{N2N}$

(15) $$F_{n}=\mathrm{Concat}\left({\hat{h}}_{\mathrm{ego}},h_{n}^{N2N}\right),$$

a two-layer MLP g(⋅) maps this fused feature to a scalar score $e_{n}=g(F_{n})$, and the goal probability over all M candidate nodes is obtained via softmax

(16) $$\theta_{n}=\frac{\exp\left(e_{n}\right)}{\sum_{m=1}^{M}\exp\left(e_{m}\right)}.$$

The output is a probability heatmap $H={\left\{\theta_{n}\right\}}_{n=1}^{M}$ over all candidate lane nodes. The estimator is trained using cross-entropy loss against a one-hot label indicating the node closest to the ground truth trajectory endpoint

(17) $$L_{\mathrm{score}}=L_{\mathrm{CE}}(\theta ,\varphi ).$$

#### 3.3.6. Candidate Screening

Before goal set optimization, topologically or physically implausible candidate nodes are filtered using the lane graph’s edge connectivity. Nodes are excluded if they are located in opposing traffic lanes, behind the current position of the target vehicle, outside the feasible driving range, or unreachable through valid $E_{\mathrm{suc}}$ or $E_{prox}$ edges from the target vehicle’s current lane. This topology-aware screening reduces the search space for subsequent optimization and prevents the generation of trajectories that violate basic traffic regulations.

#### 3.3.7. Topology-Aware Diversified Goal Selection

**Figure 4:**
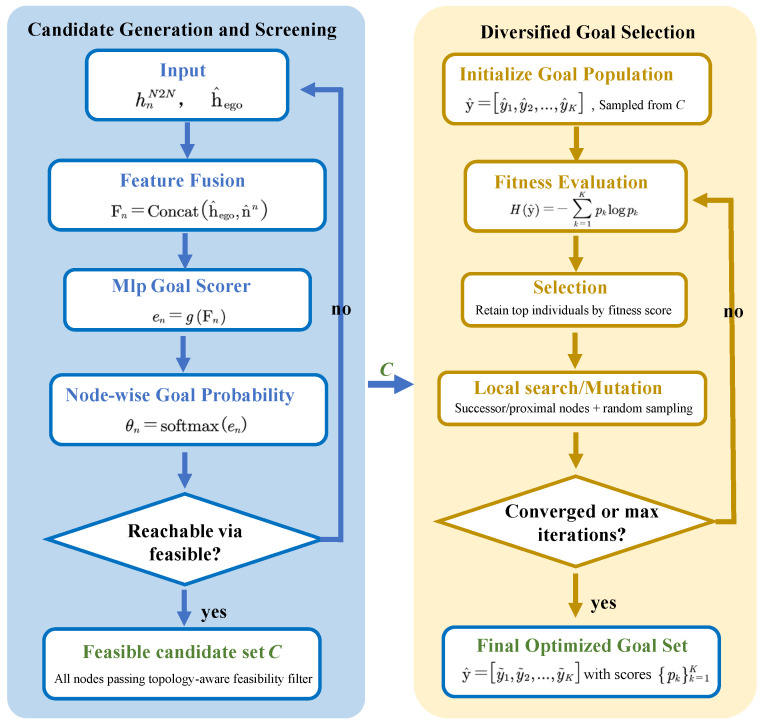
Topology-aware goal probability estimation and diversified candidate selection.

Standard thresholding-based goal selection tends to concentrate selected goals near the statistically dominant maneuver, leading to goal clustering and insufficient coverage of rare branches such as U-turns. To improve multimodal coverage, we formulate goal set selection as a combinatorial optimization problem that balances probability-weighted accuracy with spatial diversity. The full workflow of this process, from node-wise goal probability estimation and topology-aware candidate screening to diversified goal set selection, is summarized in Figure 4.

Let $\hat{y}=\left[{\hat{y}}_{1},{\hat{y}}_{2},\ldots ,{\hat{y}}_{K}\right]$ denote a candidate goal set of size K. The expected distance from all candidate nodes to the selected goal set $E\left[d\left(\hat{y},Y\right)\right]$ is

(18) $$E\left[d(\hat{y},Y)\right]=\sum_{n=1}^{M}\theta_{n}\underset{{\hat{y}}_{k}\in \hat{Y}}{\min}{\left\|{\hat{y}}_{k}-P_{n}\right\|}_{2},$$

where $P_{n}$ is the spatial position of node *n*. To discourage clustering, the entropy $H\left(\hat{y}\right)$ of the normalized goal probabilities is introduced as a diversity regularizer

(19) $$H\left(\hat{y}\right)=-\sum_{k=1}^{K}p_{k}\log p_{k},$$

where $p_{k}$ is the normalized probability assigned to selected goal ${\hat{y}}_{k}$. The full optimization objective $f\left(\hat{y}\right)$ balancing prediction accuracy and goal diversity is

(20) $$f(\hat{y})=E\left[d(\hat{y},Y)\right]-\gamma H(\hat{y}),$$

where γ>0 controls the strength of entropy regularization. The first term ensures alignment with high-probability regions, while the entropy term penalizes goal clustering and encourages the selected set to cover multiple topologically reachable exit branches.

Entropy regularization does not explicitly define intersection branches. After topology-aware screening, however, the retained candidate nodes are distributed only along reachable successor branches. If the selected goals concentrate on a dominant branch, candidate nodes on other probable branches remain far from the selected set, increasing the probability-weighted distance. Meanwhile, the entropy term discourages the selected probability distribution from concentrating on only a few candidates. Therefore, optimizing the combined objective encourages the selected goals to cover multiple reachable branches while preserving alignment with likely endpoints. Branch coverage consequently results from the joint effect of topology-aware screening, probability-weighted distance, and entropy regularization.

Because this objective is discrete and non-convex, an exhaustive search may require evaluating up to $\left(\begin{array}{c} M\\ K \end{array}\right)$ possible goal sets and becomes computationally infeasible as M increases. A greedy strategy is more efficient but makes sequential local decisions and may therefore produce spatially clustered goals. We adopt GA because its population-based optimization can directly operate on discrete candidate-node indices without requiring gradient information, while providing a balance between local exploitation and global exploration. Starting from randomly sampled goal sets drawn from the screened candidates, the algorithm iteratively refines them through two operations: (1) Local Search, which perturbs individual goals to adjacent nodes via $E_{\mathrm{suc}}$ or $E_{\mathrm{prox}}$ to improve local precision; (2) Stochastic Mutation, which randomly re-samples individual goals across the full candidate set to maintain global exploration. After optimization, the final goal set $\hat{y}=\left[{\tilde{y}}_{1},{\tilde{y}}_{2},\ldots ,{\tilde{y}}_{K}\right]$ is both high-scoring under the probability model and spatially representative of all competing exit branches. The corresponding goal scores are re-normalized to produce trajectory confidence values ${\left\{p_{k}\right\}}_{k=1}^{K}$.

### 3.4. Topology-Constrained Residual Decoder

The Topology-Constrained Residual Decoder (TCRD) transforms the selected goals and topology-propagated intent-aware scene features into physically consistent future trajectories through a two-step process: an initial GRU pass that ensures trajectory diversity, followed by a residual refinement step that enforces local lane-topology consistency at each prediction step.

To capture the variability in driving style among agents pursuing the same spatial goal—such as aggressive versus conservative acceleration profiles—a latent variable V~N(0,I) is sampled and fused with the target vehicle’s motion feature ${\hat{h}}_{\mathrm{ego}}$ and the N2N feature $g_{\mathrm{feat}}=h_{gk}^{N2N}$ of the selected goal node to initialize the decoder’s hidden state

(21) $$H_{0}=\mathrm{MLP}\left(V,{\hat{h}}_{\mathrm{ego}},g_{\mathrm{feat}}\right).$$

The decoder then employs a two-layer GRUCell to autoregressively predict an initial trajectory $\left\{{\hat{\tau }}_{t}^{k,0}\right\}$ over $T_{pred}$ steps. At each step *t*, the hidden state is updated and an initial position is regressed

(22) $$H_{t}=\mathrm{GRUCell}\left(\left[{\hat{r}}_{t-1}^{\;k},g_{\mathrm{feat}},{\hat{h}}_{\mathrm{ego}}\right],H_{t-1}\right),$$

(23) $${\hat{\tau }}_{t}^{\;k}={\hat{\tau }}_{t-1}^{\;k}+\mathrm{RegHead}(H_{t}),$$

where ${\hat{\tau }}_{t-1}^{\;k}$ is the predicted coordinate from the previous step and RegHead(⋅) is an MLP that outputs the relative displacement.

At each step t, the lane nodes nearest to the current initial predicted position ${\hat{\tau }}_{t}^{k,0}$ are identified, and their N2N features are aggregated via cross-attention to form a local topology context vector

(24) $$c_{k,t}=\mathrm{Attention}\left({\hat{\tau }}_{t}^{k,0},\left\{h_{n}^{N2N}|n\in N\left({\hat{\tau }}_{t}^{k,0}\right)\right\}\right),$$

where $N\left({\hat{\tau }}_{t}^{k,0}\right)$ denotes the set of lane nodes within a predefined distance from ${\hat{\tau }}_{t}^{k,0}$. The topology context is then fused with the initial prediction and goal feature to compute a residual correction

(25) $$\Delta {\hat{\tau }}_{k,t}=\mathrm{MLP}\left(\left[{\hat{\tau }}_{t}^{k,0},c_{k,t},g_{\mathrm{feat}}\right]\right),$$

(26) $${\hat{\tau }}_{t}^{k}={\hat{\tau }}_{t}^{k,0}+\Delta {\hat{\tau }}_{k,t}.$$

The residual formulation ensures that the initial GRU trajectory, which captures diverse temporal behavioral profiles, is preserved as the primary output, while the refinement step corrects local deviations from valid lane regions. By conditioning every refinement step on the N2N goal feature $g_{\mathrm{feat}}$ and the local topology context $c_{k,t}$, the TCRD encourages each generated trajectory to remain aligned with the selected goal and the local lane topology over the full 6-s horizon.

The distinctive feature of TCRD lies in its stepwise use of lane-topology information during decoding. It first generates a goal-conditioned base trajectory and then retrieves nearby intent-aware lane-node features at each future step to estimate a local residual correction. This residual design preserves the motion characteristics of the initial trajectory while correcting local deviations from feasible lane regions. In comparison, PGP [24] conditions predictions on sampled lane-graph traversals, whereas MTR [37] employs learnable motion queries and stacked Transformer decoder layers to refine complete trajectories. TCRD instead introduces lane topology as local feedback around the current predicted state, without requiring a predefined traversal or motion query. Together with IPIF and diversified goal selection, this design establishes a causal prediction process from intent-aware lane representation to branch-level goal inference and stepwise topology-aware trajectory refinement.

### 3.5. Learning Objective

The model is trained end-to-end with a combined loss function

(27) $$L=\beta L_{\mathrm{score}}+\mu L_{\mathrm{traj}},$$

where β and μ are weighting hyperparameters set to 1.0 in all experiments. $L_{\mathrm{score}}$ is the cross-entropy loss for goal probability estimation

(28) $$L_{\mathrm{score}}=L_{\mathrm{CE}}(\theta ,\varphi ),$$

where φ is the one-hot ground truth node label indicating the node closest to the ground truth trajectory endpoint.

$L_{\mathrm{traj}}$ is the minimum Average Displacement Error (minADE) loss, which selects the single best-matching predicted trajectory, specifically the one closest to the ground truth, and computes the average displacement error only over that trajectory

(29) $$L_{\mathrm{traj}}=\underset{k}{\min}\frac{1}{T_{\mathrm{pred}}}\sum_{t=1}^{T_{\mathrm{pred}}}\|{\hat{\tau }}_{t}^{\;k}-{\hat{\tau }}_{t}^{\mathrm{gt}}{\|}_{2},$$

where ${\hat{\tau }}_{t}^{\;gt}$ denotes the ground truth position at time step *t*. This winner-takes-all formulation encourages the model to produce at least one accurate trajectory without suppressing the diversity of the other modes.

## 4. Experiment and Result Analysis

The experimental evaluation consists of two parts. The primary comparison and ablation experiments are conducted on the XJROAD dataset, while additional external validation is performed on the public nuPlan dataset to evaluate generalization across different intersection geometries and data-collection platforms.

### 4.1. Experimental Settings

#### 4.1.1. Dataset

**Figure 5:**
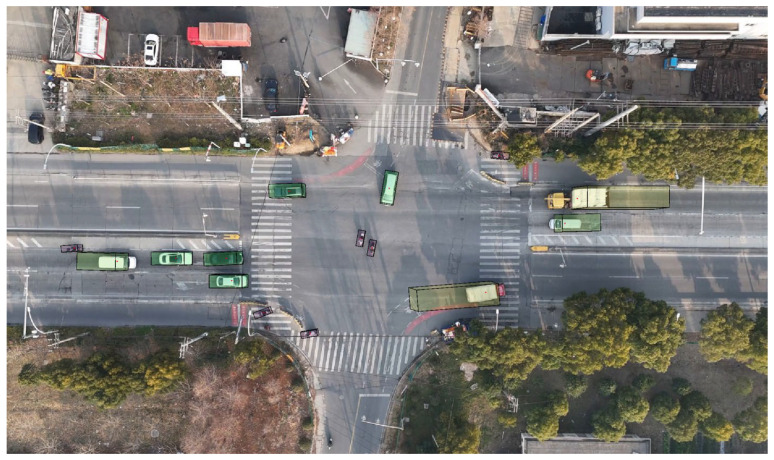
Aerial view of the XJROAD signalized intersection dataset.

This study utilizes the XJROAD dataset, which comprises 37,351 trajectories collected over a 12-h period via UAV at a two-phase signal-controlled intersection in Shanghai, China, featuring a four-lane bidirectional east–west approach and a two-lane bidirectional north–south approach (Figure 5), with over 99.8% of all traffic participants captured, including passenger cars, heavy vehicles, buses, motorcycles, and pedestrians.

To ensure temporal consistency for motion forecasting, the raw 25 Hz trajectories are downsampled to 5 Hz. The dataset is split into training, validation, and test subsets at a ratio of 7:1:2 at the trajectory level before sliding-window sample extraction, ensuring that samples generated from the same original trajectory do not appear in different subsets. A fixed-length sliding window is then applied within each subset to generate prediction samples. Each valid sample spans a total duration of 8.0 s: the first 2.0 s ($T_{\mathrm{obs}}=10$ frames) serve as the historical observation input, while the subsequent 6.0 s ($T_{\mathrm{pred}}=30$ frames) provide the ground truth for future motion prediction.

#### 4.1.2. Evaluation Metrics

We employ four metrics to assess prediction accuracy, topological compliance, and trajectory coverage.

Minimum Average Displacement Error (minADE) calculates the minimum average L2 distance between the *K* predicted trajectories and the ground truth over all $T_{\mathrm{pred}}$ steps

(30) $$\mathrm{minADE}=\underset{k\in \{1,\ldots ,K\}}{\min}\frac{1}{T_{\mathrm{pred}}}\sum_{t=1}^{T_{\mathrm{pred}}}\|{\hat{\tau }}_{t}^{\;k}-{\hat{\tau }}_{t}^{\mathrm{gt}}{\|}_{2}.$$

Minimum Final Displacement Error (minFDE) evaluates the L2 displacement at the final prediction step $T_{\mathrm{pred}}$

(31) $$\mathrm{minFDE}=\underset{k\in \{1,\ldots ,K\}}{\min}\|{\hat{\tau }}_{T_{\mathrm{pred}}}^{\;k}-{\hat{\tau }}_{T_{\mathrm{pred}}}^{\mathrm{gt}}{\|}_{2}.$$

Miss Rate (MR) measures the proportion of test samples in which no predicted trajectory falls within 2 m of the ground truth at the final step

(32) $$\mathrm{MR}=\frac{1}{N}\sum_{n=1}^{N}1\left[\underset{k}{\min}\|{\hat{\tau }}_{T_{\mathrm{pred}}}^{\;k,n}-{\hat{\tau }}_{T_{\mathrm{pred}}}^{\mathrm{gt},n}{\|}_{2}>2.0\right].$$

Off-road Rate (ORR) measures the proportion of predicted trajectory waypoints that fall outside the drivable area. A predicted point ${\hat{\tau }}_{t}^{\;k}$ is considered off-road if it falls outside the drivable area polygon defined by the lane graph. For XJROAD, the lane-centerline poses are constructed from vehicle trajectories following the trajectory-based lane-geometry construction procedure in [38]. Trajectories with similar travel directions and lane-level spatial distributions are grouped, resampled, and smoothed to obtain representative lane centerlines. Turning trajectories with the same entry–exit movement are used to construct the connection centerlines inside the intersection. The resulting centerlines are uniformly sampled into lane poses, and the regions within half of the nominal lane width from these poses jointly constitute the drivable area. Accordingly, a predicted waypoint is considered off-road when its minimum distance to all lane-centerline poses exceeds this threshold

(33) $$\mathrm{ORR}=\frac{\sum_{n=1}^{N}\sum_{k=1}^{K}\sum_{t=1}^{T_{\mathrm{pred}}}1\left[\underset{j}{\min}\|{\hat{\tau }}_{t}^{\;k,n}-p_{j}{\|}_{2}>\delta \right]}{N\cdot K\cdot T_{\mathrm{pred}}},$$

where $p_{j}$ denotes the *j*-th lane centerline pose and δ is set to half the nominal lane width. For all four metrics, lower values indicate better performance.

#### 4.1.3. Implementation Details

The proposed framework is implemented using PyTorch 1.13.1 and trained on an NVIDIA GeForce RTX 4070 GPU (NVIDIA Corporation, Santa Clara, CA, USA). The experiments are conducted in Python 3.8. Data processing and visualization are performed using NumPy 1.23.5, pandas 1.5.3, scikit-learn 1.2.2, and Matplotlib 3.7.2. The hidden dimension is set to 64 throughout all network components, and the number of predicted modes is set to K=6. All multi-head attention (MHA) modules employ 8 attention heads. The Adam optimizer is adopted with a weight decay of $1\times 10^{-4}$ and an initial learning rate of $1\times 10^{-3}$, which is decayed via cosine annealing during training. The model is trained for 64 epochs with a batch size of 32.

For the lane graph encoder, each lane node is described by P = 10 geometry poses. The N2N module uses three relation types ($E_{\mathrm{suc}},E_{\mathrm{pre}},E_{\mathrm{prox}}$) with relation-specific learnable weight matrices, applied once after A2N with hidden dimension 64. For GA-based goal optimization, the population size is set to 32, the elite ratio to 0.25, the global mutation probability to 0.05, and the maximum number of generations to 100. The TCRD generates $T_{\mathrm{pred}}=30$ steps corresponding to a 6-s prediction horizon at 5 Hz, and uses a two-layer residual refinement MLP with hidden size 64 with topology context retrieved from lane nodes within a distance threshold of 3 m from each predicted step. The total loss weighting coefficients are set to β=μ=1.0. All experiments were conducted on the same RTX 4070 GPU with batch size 32.

### 4.2. Comparison with Baseline Methods

We compare the proposed framework with ten representative baselines spanning different model families, ranging from early raster-based approaches to recent state-of-the-art Transformers, to provide a comprehensive view of performance across different design philosophies. The overall comparison results on the XJROAD dataset are reported in Table 1.

- MTP [8]: An early raster-based model that encodes BEV images via CNNs and employs multiple regression heads for multimodal trajectory generation.
- Trajectron++ [13]: A generative graph-structured recurrent model that incorporates heterogeneous agent and map data via conditional variational autoencoding.
- P2T [32]: A plan-conditioned trajectory forecasting method that generates multiple future hypotheses based on grid-based plans in unknown environments.
- MHA-JAM [11]: A model that rasterizes BEV maps and employs multi-head attention to jointly encode static scene features and surrounding agent interactions for trajectory prediction.
- PGP [24]: A goal-oriented model that predicts paths on a lane graph using selective traversals conditioned on a behavior-cloning policy.
- HiVT [39]: A hierarchical vectorized Transformer that captures local and global context through translation-invariant spatiotemporal embeddings.
- MTR [37]: A motion-query based Transformer that refines trajectory predictions via global intention localization and local movement refinement.
- QCNet [40]: A query-centric framework designed for spatiotemporal invariance and high-efficiency scene modeling.
- Forecast-MAE [41]: A masked autoencoder model that leverages self-supervised pre-training to capture deep temporal and social dependencies.
- MTR++ [42]: An advanced motion Transformer incorporating symmetric scene modeling and improved intention querying for complex scenarios.

The results reveal a clear performance progression across model families. Raster-based methods and the IRL-based P2T generally perform below lane-graph and Transformer-based methods on XJROAD. This may be partly attributed to their limited ability to explicitly encode lane topology in branching intersection scenarios. Their higher ORR values further suggest that unconstrained spatial representations are less effective in keeping predictions within valid lane regions. Among these methods, P2T shows a relative advantage over purely raster-based approaches because its policy-based sampling at least partially respects scene structure, but it still falls short of models that incorporate explicit lane graph connectivity.

Lane-graph-based and Transformer-based methods form a tightly competitive group. The proposed method achieves the best performance across all four metrics. Compared to PGP, the strongest lane-graph baseline, the proposed method shows improvements of 15.6%, 22.4%, and 43.9% on minADE, minFDE, and MR, respectively. The gain in MR is particularly pronounced, suggesting that the proposed method improves coverage of rare but safety-critical maneuvers that simpler goal selection methods tend to miss. Compared to MTR++, the strongest Transformer-based baseline, the proposed method improves MR by 20.7% and achieves the lowest ORR (0.024)—less than half of MTR++’s ORR (0.054)—demonstrating that explicit lane-topology encoding through IPIF and topology-constrained decoding through TCRD provide strong complementary benefits for both prediction accuracy and topological consistency.

### 4.3. Impact of Prediction Time Horizon

To evaluate how prediction performance changes across forecasting horizons, we analyze minADE, minFDE, and MR as a function of the prediction horizon T for the proposed method and the three strongest baselines (MTR++, Forecast-MAE, QCNet), with *K* = 6 fixed.

**Figure 6:**
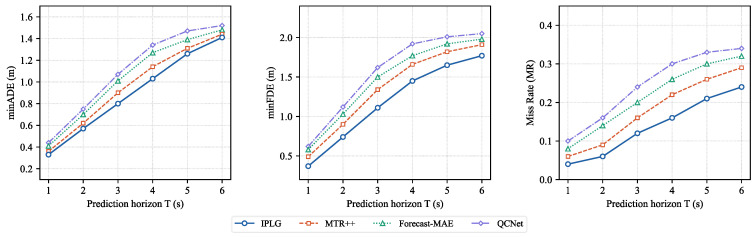
Prediction performance of IPLG and three baselines as a function of prediction time horizon T.

As shown in Figure 6, at short prediction horizons (T = 1 s), IPLG already shows a noticeable advantage over all baselines, particularly in minADE, suggesting that the intent-aware lane features produced by IPIF provide effective constraint even at very short ranges. As T increases, prediction errors rise progressively across all methods, and the performance gap between IPLG and the baselines widens consistently. IPLG consistently achieves the lowest minADE, minFDE, and MR across all horizons. The advantage is particularly pronounced in Miss Rate at longer horizons (T = 5–6 s), suggesting that the intent-aware lane features produced by IPIF and the diversified goal set from the proposed goal selection strategy contribute to more robust long-term multimodal coverage.

### 4.4. Impact of the Number of Predicted Modes

**Figure 7:**
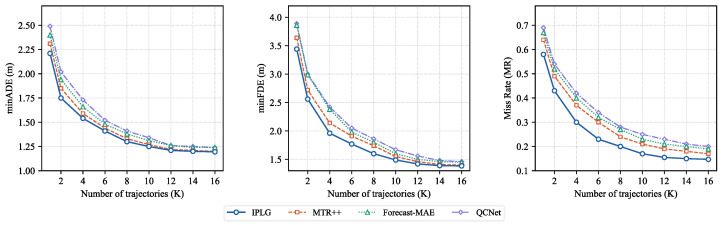
Prediction performance of IPLG and three baselines as a function of the number of predicted modes *K*.

We investigate how prediction performance varies with the number of predicted modes K∈{1,2,4,6,8,10,12,16}, comparing the proposed method against MTR++, Forecast-MAE, and QCNet with T = 6 s fixed. Results are shown in Figure 7.

All models exhibit the weakest performance at *K* = 1 and improve monotonically as *K* increases, with the rate of improvement decelerating beyond *K* = 6. IPLG maintains a consistent advantage across all *K* values, with the margin most pronounced at small *K*. This reflects a key property of IPLG’s diversity-aware goal selection: by encouraging spatial diversity among selected goals, IPLG generates modes that tend to span multiple exit branches even at small *K*, whereas methods without entropy regularization tend to concentrate multiple modes on the same dominant branch. Among the three metrics, the advantage of IPLG is most persistent in Miss Rate, where the gap over baselines remains visible even at *K* = 16, suggesting that diversity-aware goal selection contributes not only to displacement accuracy but also to coverage of rare maneuver branches. As *K* increases beyond 10, the displacement error gap between methods narrows, indicating that explicit diversity enforcement is most impactful in the practically relevant regime of small-to-moderate mode counts.

### 4.5. Ablation Studies

We conduct four ablation analyses on the XJROAD dataset with K=6 and T=6 fixed, including progressive interaction-component ablation, N2N propagation-depth analysis, goal-selection ablation, and decoder ablation. All variants are trained under identical settings. The results are reported in Table 2, Table 3, Table 4 and Table 5.

#### 4.5.1. Intent-Propagated Interaction Flow

Table 2 reports the progressive contribution of each interaction stage. Introducing N2A alone (Variant 1) yields improvements of 5.8%, 5.9%, and 11.9% in minADE, minFDE, and MR over the base encoder, confirming that aggregating road-context information from nearby lane nodes provides a useful foundation for intent-aware prediction. Adding A2A yields further improvements of 6.2%, 5.3%, and 8.1% in minADE, minFDE, and MR respectively, reflecting enhanced social awareness through agent-to-agent context aggregation. The introduction of A2N brings a particularly notable improvement in MR, reducing it from 0.34 to 0.30, which confirms its importance for covering rare maneuvers. By propagating agent kinematic cues back into lane nodes, the model resolves the semantic ambiguity of divergence-point nodes and assigns nonzero probability to all reachable exit branches. A2N also leads to a clear ORR reduction from 0.038 to 0.031, indicating that intent-aware lane representations improve topological consistency. Adding N2N brings further gains of 4.1% in minADE, 7.3% in minFDE, and 23.3% in MR, with ORR further reduced to 0.024, confirming that propagating intent semantics along lane topology after A2N enables downstream branch nodes to inherit intention context from upstream divergence points, improving both goal probability estimation and trajectory lane alignment across the full lane graph.

**Table 2:** Ablation study of the Intent-Propagated Interaction Flow (*K* = 6).

Variant	N2A	A2A	A2N	N2N	minADE	minFDE	MR	ORR
1	√	-	-	-	1.62	2.08	0.37	0.043
2	√	√	-	-	1.52	1.97	0.34	0.038
3	√	√	√	-	1.47	1.91	0.30	0.031
4	√	√	√	√	1.41	1.77	0.23	0.024

To further investigate whether deeper topological propagation improves intent representation, we evaluate N2N configurations with zero, one, two, and three propagation layers. All variants use the complete N2A–A2A–A2N interaction flow and share identical feature dimensions, goal-selection settings, decoder configurations, and training protocols.

**Table 3:** Ablation study on the number of N2N propagation layers (*K* = 6).

N2N Layers	minADE	minFDE	MR	ORR
0	1.47	1.91	0.30	0.031
1	1.41	1.77	0.23	0.024
2	1.44	1.83	0.25	0.027
3	1.49	1.90	0.29	0.030

As shown in Table 3, removing N2N leads to clear performance degradation across all four metrics, confirming the necessity of propagating agent-conditioned lane-node features along the lane topology. The single-layer configuration achieves the best overall performance. Increasing the propagation depth to two or three layers provides no further improvement and instead results in slightly higher displacement errors, MR, and ORR. This suggests that repeated message aggregation may mix representations from competing intersection branches and gradually weaken branch-specific semantics through over-smoothing. Moreover, uncertain intention cues may be propagated to increasingly distant nodes as the propagation depth increases. Therefore, a single N2N layer provides the best balance between effective local intent propagation and preservation of branch-level discrimination.

The degradation observed with deeper N2N configurations also indicates that repeated topological propagation may amplify uncertain intention cues and mix features from competing branches. Therefore, the single-layer configuration not only achieves the best prediction performance but also provides a practical mechanism for limiting error propagation.

#### 4.5.2. Goal Set Prediction Strategy

Table 4 compares five goal selection strategies with progressively added components. Simple thresholding yields only marginal gains over the base encoder, confirming that naive threshold-based goal selection is prone to mode collapse and insufficient for covering diverse exit branches. Adding topology-aware filtering substantially improves all metrics, with minADE dropping from 1.73 to 1.57, demonstrating that excluding physically infeasible candidates is a necessary prerequisite for meaningful goal diversity. The GA-only variant slightly improves minADE compared with topology filtering alone, but its minFDE and MR remain worse, suggesting that GA optimization without entropy regularization mainly refines local endpoint precision but does not sufficiently improve branch coverage. However, its MR (0.34) is notably worse than the Entropy-only variant (0.30), confirming that GA without entropy regularization still converges toward locally optimal solutions that concentrate goals in high-probability regions and fails to adequately cover minority exit branches. The entropy regularization term is the primary driver of MR improvement: Comparing Entropy-only with GA-only shows that entropy regularization is more effective for MR reduction, decreasing MR from 0.34 to 0.30. Moreover, compared with the full ME + GA variant, removing entropy leads to a clear MR degradation from 0.23 to 0.34. The full ME + GA combination achieves the best performance across all metrics (1.41/1.77/0.23), confirming that prediction accuracy and mode diversity are complementary objectives that must be jointly optimized. The ORR column further shows that topology-aware filtering is the primary driver of off-road reduction (0.051 → 0.038), while the full ME + GA combination achieves the lowest ORR (0.024) by selecting goals that are both diverse and well-aligned with feasible lane regions.

**Table 4:** Ablation study of the goal selection strategy (*K* = 6).

Goal Selection	Topology Filter	Entropy Reg	GA	minADE	minFDE	MR	ORR
Thresholding	-	-	-	1.73	2.16	0.41	0.051
Topology filter	√	-	-	1.57	1.96	0.39	0.038
Entropy only	√	√	-	1.52	1.94	0.30	0.033
GA only	√	-	√	1.55	1.98	0.34	0.036
ME + GA	√	√	√	1.41	1.77	0.23	0.024

**Figure 8:**
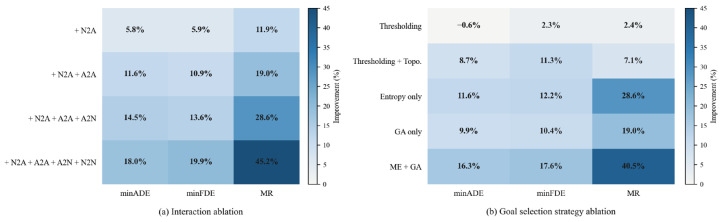
Ablation study results. (**a**) Progressive contribution of each component in the Interaction mechanism. (**b**) Comparison of goal selection strategies.

To further summarize the relative contribution of different components, Figure 8 visualizes the percentage improvements of all ablation variants over the base encoder in terms of minADE, minFDE, and MR. In panel (a), the color intensity increases progressively from top to bottom, with the MR column showing the steepest gradient, confirming that the IPIF contributes most significantly to multimodal branch coverage. The A2N variant shows a clear increase in MR improvement compared with the preceding variants, reinforcing the importance of closing the agent-to-node feedback loop. The N2N row achieves the darkest coloring across all three columns, visually confirming that topology propagation provides the largest cumulative gain among all interaction stages. In panel (b), the difference between the Thresholding and ME + GA rows indicates the cumulative benefit of the full goal selection pipeline. The MR column again shows the strongest color gradient, indicating that goal diversity enforcement has a more pronounced effect on miss rate than on displacement errors. The Entropy-only row achieves notably darker coloring in MR than the GA-only row, visually confirming that entropy regularization is the primary driver of branch coverage while GA contributes more to displacement precision.

**Figure 9:**
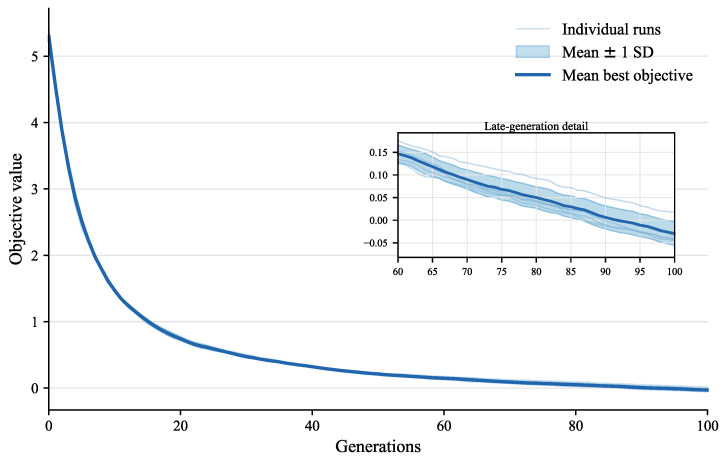
Convergence behavior of GA-based goal-set optimization on the validation set. (The solid line and shaded region represent the mean best objective and one standard deviation over five independent runs).

We further evaluate the convergence behavior and stability of GA-based goal optimization on a fixed validation subset. The optimization is independently repeated five times using different random seeds. As shown in Figure 9, the mean best objective decreases rapidly during the early generations and then improves more gradually. More than 95% of the total objective reduction is achieved within the first 60 generations, after which the optimization approaches a stable solution. The narrow variation among the five independent runs indicates limited sensitivity to random initialization and confirms the convergence stability of the adopted GA configuration. Since the entropy regularization term is subtracted from the probability-weighted distance term, the objective value may become slightly negative.

#### 4.5.3. Ablation Study of the Topology-Constrained Residual Decoder

Table 5 compares three decoder configurations to isolate the contribution of the residual refinement step. The plain GRU decoder generates trajectories conditioned on the selected goal without any topological feedback during decoding. Adding latent variable sampling improves trajectory diversity, with MR dropping from 0.28 to 0.26, but does not substantially reduce ORR, as the decoder still lacks stepwise lane-node context. The full TCRD achieves the best performance across all metrics, with the most pronounced improvement in ORR (0.024 vs. 0.035, a 31.4% reduction). This indicates that retrieving local intent-aware lane-node context at each prediction step helps correct local deviations from feasible lane regions, thereby reducing ORR without degrading multimodal coverage.

**Table 5:** Ablation study of the Topology-Constrained Residual Decoder (*K* = 6).

Decoder	minADE	minFDE	MR	ORR
GRU	1.51	1.91	0.28	0.043
GRU + latent	1.47	1.86	0.26	0.035
TCRD	1.41	1.77	0.23	0.024

### 4.6. Trajectory Diversity Analysis

To quantify the extent to which the proposed method addresses mode collapse, we evaluate four diversity metrics on the XJROAD test set for *K* = 6 and *K* = 10: the number of distinct final lanes covered, the variance of final yaw angle ($\sigma_{\mathrm{yaw}}^{2}$), the variance of average speed ($\sigma_{\mathrm{speed}}^{2}$), and the variance of average acceleration ($\sigma_{\mathrm{acc}}^{2}$). Higher values indicate greater trajectory diversity. The trajectory diversity comparison on the XJROAD dataset is reported in Table 6.

The proposed method achieves 1.63 distinct final lanes at *K* = 6, exceeding PGP (1.27, +28.3%) and MTR++ (1.41, +15.6%). The ablation row “IPLG w/o ME” achieves only 1.44 distinct lanes—close to MTR++—confirming that the lateral diversity gain is specifically attributable to the topology-aware diversified goal selection module. The proposed method also shows higher longitudinal diversity: at *K* = 6, $\sigma_{\mathrm{speed}}^{2}$ and $\sigma_{\mathrm{acc}}^{2}$ improve by 60.5% and 48.1% over PGP, respectively. This may be partly related to the latent variable sampling in the TCRD, which allows different temporal behavioral profiles under similar spatial intentions.

### 4.7. Computational Efficiency and Complexity Analysis

To evaluate the computational efficiency of IPLG, Table 7 reports the number of trainable parameters and the average inference time of each component for a representative scene containing 10 surrounding agents. Data loading and map preprocessing are excluded from the inference time.

As shown in Table 7, IPLG contains 1.65 million trainable parameters and requires 215 ms per scene. Goal set prediction accounts for most of the inference time because of the iterative GA optimization, although the GA itself introduces no trainable parameters.

Let $E_{AN}$ denote the set of local agent–lane-node associations and d the hidden feature dimension. Using the agent set A and lane-edge set E defined in Section 3.1, the computational complexity of IPIF is approximately.

(34) $$O\left((|E_{\mathrm{AN}}\left|+\right|A{|}^{2}+|E|)d\right),$$

where the three terms correspond to agent–lane interaction, agent–agent interaction, and lane-topology propagation, respectively. Using the previously defined K prediction modes and T future steps, the complexity of TCRD is approximately $O(KTd^{2}).$

Let $N_{\mathrm{iter}}$ and $N_{\mathrm{pop}}$ denote the number of GA iterations and population size, respectively. Using the previously defined number M of candidate goals, the complexity of GA-based goal selection is approximately $O(N_{\mathrm{iter}}N_{\mathrm{pop}}KM).$

Overall, GA-based goal selection is the main computational bottleneck of IPLG. The current implementation processes approximately 4.7 scenes per second. Although this performance is sufficient for offline evaluation, further acceleration is required for high-frequency real-time deployment. Possible alternatives include greedy selection, beam search, determinantal point processes, differentiable Top-*K* relaxation, and learned goal-set prediction. Greedy and beam-search methods may reduce inference latency but are more sensitive to selection order and pruning decisions and provide limited global exploration. Differentiable and learned selectors support end-to-end optimization but require additional architectural design and training while explicitly preserving topological feasibility. In addition, parallel population evaluation and adaptive early stopping could accelerate the current GA implementation. These directions will be investigated in future work to reduce the latency of goal-set selection.

### 4.8. Qualitative Analysis

**Figure 10:**
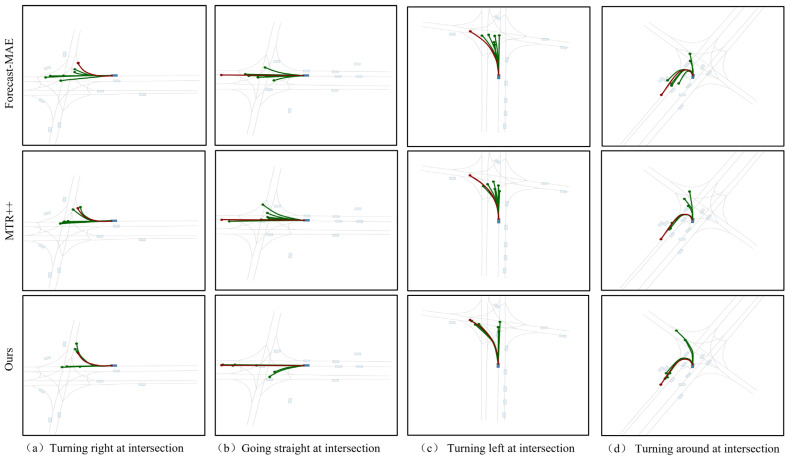
Qualitative results of IPLG on the XJROAD dataset. The red line denotes the ground-truth future trajectory, the green lines denote the predicted trajectories, and the blue rectangle denotes the target vehicle. The light-blue rectangles represent surrounding vehicles, and the gray lines indicate lane centerlines.

We qualitatively compare the proposed method against the two strongest baselines, MTR++ and Forecast-MAE, on the XJROAD dataset. Figure 10 presents prediction results across four representative maneuver scenarios—right turn, straight-going, left turn, and U-turn. Green trajectories represent the *K* predicted modes and red trajectory represents the ground truth, with the target vehicle shown in blue.

In the right-turn scenario (a), Forecast-MAE generates trajectories that are predominantly directed straight ahead, with only weak coverage of the right-turn branch. MTR++ captures the right-turn tendency, but its predicted trajectories show noticeable deviations from the turning lane centerline. In contrast, IPLG covers both the straight-through and right-turn branches, with the right-turn mode more closely aligned with the ground-truth trajectory. This indicates that the proposed intent-propagated lane representation helps preserve plausible branch-level alternatives even when one maneuver is dominant.

In the straight-going scenario (b), all three methods capture the dominant straight-ahead intention to some extent. However, Forecast-MAE and MTR++ generate several trajectories that deviate from the feasible driving corridor and are less consistent with the lane geometry. In contrast, IPLG produces trajectories that remain better aligned with the straight-through lane while preserving reasonable multimodal coverage of adjacent feasible branches. This suggests that the topology-aware candidate screening and TCRD refinement help suppress geometrically implausible predictions.

In the left-turn scenario (c), the left-turn maneuver requires accurate branch-level intention discrimination and consistency with the curved lane geometry. Forecast-MAE does not adequately cover the left-turn branch. MTR++ produces one trajectory with a left-turn tendency, but most of its predicted modes are not well aligned with the left-turn direction and fail to consistently cover the intended branch. In contrast, IPLG generates three geometrically consistent left-turn trajectories while preserving plausible alternatives along the feasible lane topology. This can be attributed to the IPIF module, where A2N injects agent kinematic cues into divergence-point lane nodes and N2N further propagates the resulting intent-aware semantics along the lane topology, enabling more reliable probability assignment to the left-turn exit.

In the U-turn scenario (d), the maneuver is strongly constrained by sharp curvature and lane topology. Forecast-MAE produces several U-turn-like trajectories, but their turning angles are geometrically implausible and deviate from the feasible turning corridor. MTR++ generates three reasonable U-turn trajectory, but some of its additional modes still violate lane-topology constraints. In contrast, IPLG produces a more coherent set of predictions, with the U-turn mode closely following the feasible lane geometry and the remaining modes staying within plausible driving regions. This result highlights the benefit of combining topology-aware candidate screening, entropy-regularized goal optimization, and TCRD refinement, which together preserve reachable U-turn candidates, encourage diverse branch coverage, and correct local deviations from valid lane regions.

Overall, these qualitative results indicate that IPLG achieves a favorable balance between prediction accuracy, multimodal coverage, and topological consistency. The comparison further demonstrates the complementary benefits of the IPIF encoder, the topology-aware goal selection module, and the TCRD in handling complex intersection maneuvers.

### 4.9. External Validation on nuPlan

To further evaluate the generalization and reproducibility of IPLG, we conduct additional experiments on the public nuPlan dataset [43]. nuPlan is a large-scale, industry-grade autonomous-driving dataset containing approximately 1282 h of real-world driving data collected from Las Vegas, Boston, Pittsburgh, and Singapore. It provides continuous object tracks, traffic-light states, and detailed HD map information for diverse urban driving scenarios. These continuous vehicle tracks can be divided into historical observations and future labels to construct a trajectory-prediction task. Moreover, the dataset contains diverse scenarios involving lane changes, vehicle merging, intersection navigation, and multi-agent interactions. Therefore, nuPlan provides a suitable public benchmark for evaluating the generalization of the proposed topology-aware intent representation and trajectory-decoding method across different road geometries and data-collection platforms.

For data preprocessing, vehicle tracks containing complete observation and prediction periods are extracted from intersection-related scenarios. Consistent with the XJROAD experiments, the model observes 2 s of historical motion and predicts the subsequent 6 s. Only vehicle tracks with complete temporal observations are retained. Local lane and lane-connector geometries are extracted from the official nuPlan HD map to provide road-geometry and drivable-area information. Agent trajectories and map elements are subsequently transformed into a target-centered coordinate system. All methods use the same input and output horizons and generate six future trajectory modes. The evaluation adopts minADE, minFDE, MR, and ORR, following the definitions presented in Section 4.1.2.

As shown in Table 8, IPLG achieves the best performance across all four metrics. Compared with MTR++, the strongest baseline, IPLG reduces minADE and minFDE by 4.9% and 8.6%, respectively. It also reduces MR and ORR by 13.3% and 28.6%, respectively. These improvements demonstrate that IPLG improves both long-term prediction accuracy and consistency with feasible driving regions on the nuPlan dataset.

Multi-Kinematic produces substantially higher displacement errors and MR because it relies primarily on short-term kinematic extrapolation and cannot explicitly distinguish competing intersection branches. Its ORR remains relatively moderate because an incorrectly selected trajectory may still remain within the overall drivable area despite deviating considerably from the ground-truth destination. Trajectron++ improves prediction accuracy through agent-interaction modeling but lacks explicit lane-topology constraints. PGP, GameFormer, and MTR++ achieve progressively better performance by incorporating lane-graph reasoning or attention-based interaction modeling. Nevertheless, their higher MR and ORR values indicate that branch-level intention coverage and topological consistency remain challenging in complex intersection scenarios.

**Figure 11:**
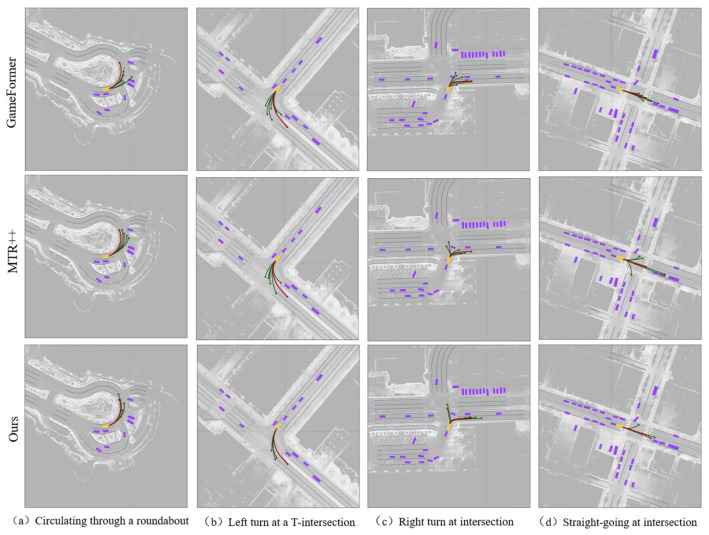
Qualitative trajectory-prediction results on four nuPlan intersections. The red line denotes the ground-truth future trajectory, the green lines denote the predicted trajectories, the yellow rectangle denotes the target vehicle, the purple rectangles represent surrounding vehicles, and the gray lines indicate lane centerlines.

Figure 11 presents qualitative comparisons among GameFormer, MTR++, and IPLG across four representative nuPlan intersection geometries. From left to right, the four columns show a roundabout, a T-intersection, a multi-phase signalized intersection, and a four-way intersection. These scenarios contain curved movement, turning, straight driving, and competing downstream branches.

As illustrated in Figure 11, the baseline methods generally capture the dominant movement direction but may generate trajectories that concentrate on similar branches or deviate from feasible lane regions. In contrast, IPLG produces trajectories that more closely follow the local lane geometry while retaining feasible alternatives at intersection divergence points. This advantage is particularly evident in the roundabout and signalized-intersection scenarios, where future motion is strongly constrained by curved lane connections and competing downstream branches.

Overall, the quantitative and qualitative results on nuPlan demonstrate that the proposed model maintains competitive prediction accuracy and generates reasonable trajectories across different intersection geometries. These results further support its effectiveness and generalizability on an external public dataset.

## 5. Conclusions

This paper presents IPLG, an Intent-Propagated Lane Graph framework with Topology-Constrained Residual Decoding for multimodal vehicle trajectory prediction at urban intersections. The framework addresses two core challenges that are particularly acute in intersection scenarios: the semantic ambiguity of divergence-point lane nodes under competing agent intentions, and the tendency of standard decoders to generate trajectories that deviate from valid lane regions.

IPLG tackles these challenges through three tightly coupled components. In the interaction reasoning stage, the Intent-Propagated Interaction Flow (IPIF) models closed-loop agent–lane dependencies through four sequential stages that collectively transform static lane-node features into topology-propagated, intent-aware representations. N2A aggregates road context into agent representations; A2A captures social dynamics among neighboring agents; A2N injects agent kinematic cues back into lane nodes, resolving the semantic ambiguity of divergence-point nodes whose topological role depends on surrounding traffic intent; and N2N propagates the resulting intent-aware features along lane topology, enabling downstream branch nodes to inherit intention context from upstream divergence points before goal selection. Ablation results confirm that the four-stage IPIF contributes most significantly to multimodal branch coverage, with MR and ORR improvements accumulating progressively across all stages. In the goal inference stage, the proposed goal selection module combines entropy regularization with combinatorial optimization to select a spatially diverse goal set from topology-aware candidate nodes, addressing mode collapse without requiring manual anchor design. Ablation results demonstrate that entropy regularization is the primary driver of MR improvement, while the combinatorial optimization contributes primarily to spatial precision, and their combination achieves the best balance across all metrics. In the decoding stage, the Topology-Constrained Residual Decoder (TCRD) generates diverse goal-conditioned GRU trajectories and applies residual refinement using nearby lane-node context at each prediction step, substantially reducing off-road violations without constraining trajectory diversity, as confirmed by ablation results showing the most pronounced improvement in ORR among all three decoder configurations.

Experiments on the XJROAD dataset confirm that IPLG outperforms competitive baselines across all four evaluation metrics, with particularly clear advantages in Miss Rate and Off-road Rate. Qualitative results further show that IPLG provides stable coverage of representative maneuvers, including right turns, straight driving, left turns, and U-turns, while maintaining consistency with lane topology. Additional quantitative and qualitative validation on the public nuPlan dataset demonstrates that the proposed model maintains competitive prediction accuracy across roundabouts, T-intersections, multi-phase signalized intersections, and four-way intersections. These results support the effectiveness and generalizability of IPLG across different road geometries and data-collection platforms.

This study has several limitations that suggest directions for future work. First, the current evaluation is conducted offline using recorded trajectories and preprocessed lane-graph information. Before deployment in operational autonomous-driving systems, the robustness of IPLG to perception noise, tracking errors, map inaccuracies, and closed-loop interactions requires further investigation. Second, the optimization-based goal-selection process introduces non-negligible inference latency, which may limit real-time deployment in resource-constrained systems; future work could explore differentiable or learned approximations for diversified goal selection. Third, IPLG currently treats all agent types uniformly; incorporating agent-specific motion and interaction characteristics for vehicles, pedestrians, and cyclists could improve its applicability to complex mixed-traffic environments.

## Figures and Tables

**Table 1:** Overall comparison with baseline methods on XJROAD (*K* = 6).

Method	minADE	minFDE	MR	ORR
MTP	2.11	2.96	0.57	0.120
Trajectron++	2.01	2.79	0.54	0.105
MHA-JAM	1.88	2.54	0.49	0.091
P2T	1.73	2.41	0.45	0.075
PGP	1.67	2.28	0.41	0.065
HiVT	1.61	2.18	0.39	0.078
MTR	1.57	2.10	0.37	0.069
QCNet	1.52	2.05	0.34	0.065
Forecast-MAE	1.48	1.98	0.32	0.067
MTR++	1.44	1.91	0.29	0.054
IPLG (ours)	1.41	1.77	0.23	0.024

**Table 6:** Trajectory diversity comparison on XJROAD.

Method	*K*	Distinct Lanes	$\sigma_{\mathrm{yaw}}^{2}$	$\sigma_{\mathrm{speed}}^{2}$	$\sigma_{\mathrm{acc}}^{2}$
PGP	6	1.27	0.10	2.91	5.07
MTR++	6	1.41	0.12	3.51	5.99
IPLG w/o ME	6	1.44	0.13	3.68	6.19
IPLG	6	1.63	0.15	4.67	7.51
PGP	10	1.42	0.13	3.87	6.30
MTR++	10	1.55	0.15	4.46	7.15
IPLG w/o ME	10	1.58	0.14	4.27	6.84
IPLG	10	1.76	0.17	5.49	8.39

**Table 7:** Parameter count and inference time of different IPLG components.

Component	Params (M)	Inference Time (ms)
Agent and lane-node encoders	0.28	8
IPIF	0.75	24
Goal set prediction (GA optimization)	0.20	138
TCRD	0.42	45
Total	1.65	215

Note: M: million; ms: milliseconds.

**Table 8:** Quantitative comparison on the nuPlan dataset (*K* = 6).

Method	minADE	minFDE	MR	ORR
Multi-Kinematic	3.48	9.19	0.83	0.074
Trajectron++	2.32	3.34	0.52	0.085
PGP	1.91	2.72	0.42	0.073
GameFormer	1.72	2.39	0.35	0.065
MTR++	1.63	2.22	0.30	0.049
IPLG (ours)	1.55	2.03	0.26	0.035

## Data Availability

The data presented in this study are available on request from the corresponding author. The data are not publicly available due to privacy.
